# Development and validation of a novel T cell proliferation-related prognostic model for predicting survival and immunotherapy benefits in melanoma

**DOI:** 10.18632/aging.204748

**Published:** 2023-05-24

**Authors:** Jiajie Chen, Daiyue Wang, Shixin Chan, Qingqing Yang, Chen Wang, Xu Wang, Rui Sun, Yu Gui, Shuling Yu, Jinwei Yang, Haoxue Zhang, Xiaomin Zhang, Kechao Tang, Huabing Zhang, Shengxiu Liu

**Affiliations:** 1 Department of Dermatology, The First Affiliated Hospital of Anhui Medical University, Hefei, Anhui 230022, China; 2 Key Laboratory of Dermatology, Anhui Medical University, Ministry of Education, Hefei, Anhui 230022, China; 3 Inflammation and Immune-Mediated Diseases Laboratory of Anhui Province, Hefei, Anhui 230022, China; 4 Department of General Surgery, The First Affiliated Hospital of Anhui Medical University, Hefei, Anhui 230022, China; 5 Affiliated Chuzhou Hospital of Anhui Medical University, The First People’s Hospital of Chuzhou, Chuzhou, Anhui 230022, China; 6 Department of Biochemistry and Molecular Biology, Metabolic Disease Research Center, School of Basic Medicine, Anhui Medical University, Hefei, Anhui 230022, China

**Keywords:** melanoma, T cell proliferation, prognosis, immune landscape, immunotherapy

## Abstract

Background: T cell plays a crucial role in the occurrence and progression of Skin cutaneous melanoma (SKCM). This research aims to identify the actions of T cell proliferation-related genes (TRGs) on the prognosis and immunotherapy response of tumor patients.

Method: The clinical manifestation and gene expression data of SKCM patients were obtained from The Cancer Genome Atlas (TCGA) and Gene Expression Omnibus (GEO) databases. T cell proliferation-related molecular subtypes were identified utilizing consensus clustering. Subsequently, Cox and Lasso regression analysis was conducted to identify six prognostic genes, and a prognostic signature was constructed. A series of experiments, such as qRT-PCR, Western blotting and CCK8 assay, were then conducted to verify the reliability of the six genes.

Results: In this study, a grading system was established to forecast survival time and responses to immunotherapy, providing an overview of the tumoral immune landscape. Meanwhile, we identified six prognostic signature genes. Notably, we also found that C1RL protein may inhibit the growth of melanoma cell lines.

Conclusion: The scoring system depending on six prognostic genes showed great efficiency in predicting survival time. The system could help to forecast prognosis of SKCM patients, characterize SKCM immunological condition, assess patient immunotherapy response.

## INTRODUCTION

Skin cutaneous melanoma (SKCM) is a common type of skin cancer, and its incidence has increased rapidly in recent decades [1]. SKCM develops from the malignant transformation of melanocytes in the basal layer of the skin epidermis, and is highly invasive. It is the most deadly skin cancer worldwide, and the number of deaths caused by SKCM is still increasing year by year [2]. The main factors that induce melanoma are environmental factors, genetic factors and immune factors [3]. At present, surgery is the main treatment for SKCM, but the continuously improved surgical methods have not significantly improved the disease-free survival (DFS) and overall survival (OS) of the disease [4]. Immunotherapy has been widely used in melanoma worldwide, and a large number of clinical trial results have highlighted the efficacy of immunotherapy for advanced metastatic melanoma. However, the drug resistance of patients and the toxicity of some immunotherapy drugs determine that the current treatment methods still have certain limitations [5–7]. Therefore, it is necessary to explore the factors affecting the prognosis of the disease and more effective treatment methods.

T lymphocytes, especially their antigen-directed cytotoxicity, have become a central focus of the immune system in cancer prevention [8]. Immunotherapeutics are the fastest-growing drug class and have a major impact on cancer treatment and human health [9]. Adoptive T-cell (ATC) treatment, in which autologous or allogeneic T cells are introduced into patients, has had encouraging results in recent years. *In vitro* expansion of tumor-specific T cells is crucial for facilitating the development of engineered lymphocytes [10]. T cell activity is controlled by several negative regulators that act as ‘checkpoint molecules’ [11]. Immune checkpoint inhibitors (ICIs) are antibodies specifically targeting the immunomodulatory molecules cytotoxic T lymphocyte-associated protein 4 (anti-CTLA-4) and programmed cell death protein 1 (anti-PD-1) and have been authorized by some official regulatory agencies. These two inhibitors play a key part in the treatment of SKCM [12, 13]. Notably, melanoma is an ideal model to investigate various immunotherapies, including checkpoint inhibitors, anticancer vaccines, and engineered chimeric antigen receptor T cells [14–16]. TME is crucial for tumor formation and growth, and it could influence tumor response to immunotherapy [17]. TME contains immunological and inflammatory cells, extracellular matrix, and released cytokines [18]. Previous studies reported that the tumor mutation burden (TMB) could be utilized to forecast response to immunotherapy [19].

A recent study discovered a total of 33 genes called T cell proliferation-related genes (TRGs), which could drive T cell proliferation, promote proinflammatory cytokine secretion and increase the expression of activation markers [20]. However, the effects of these T cell proliferation-related genes (TRGs) on the prognosis and treatment responses of patients with SKCM remain unclear.

In this study, the expression profiles of TRGs were evaluated comprehensively, providing an overview of tumoral immunological landscape. First, SKCM patients were stratified into two distinct T cell proliferation molecular subtypes based on the TRG expression. Then, these patients were split into 2 gene clusters according to differentially expressed genes (DEGs). A TRG_score model based on six prognostic signature genes was designed to forecast prognosis and response to immunotherapy. Moreover, qRT-PCR was utilized to measure the six signature genes expressions in two SKCM cell lines and one corresponding normal melanocyte line to identify the efficiency of these genes.

## RESULTS

### Genetic and transcriptional changes of TRGs in SKCM

**Figure 1:**
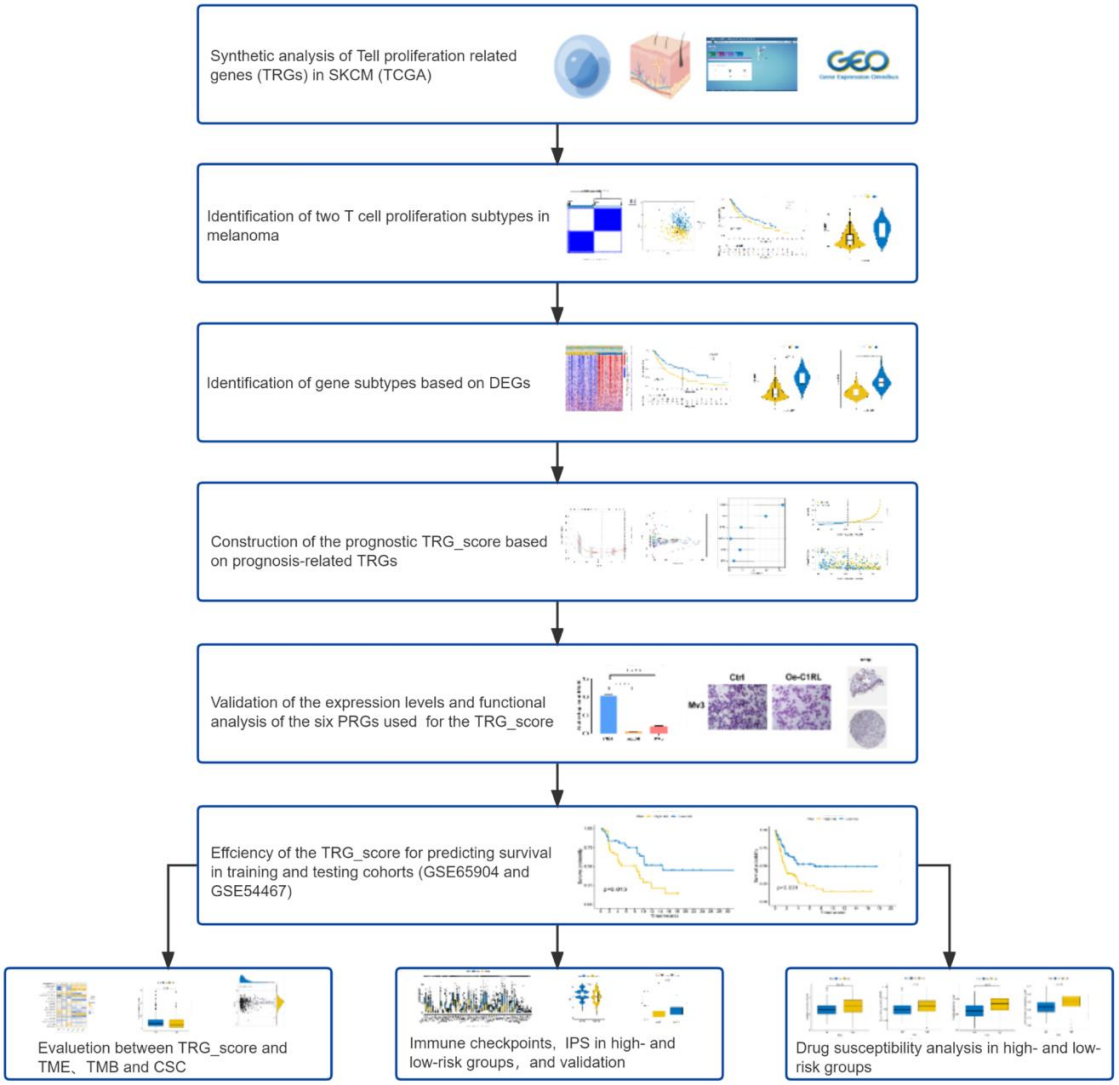
Flow diagram of the study.

**Figure 2:**
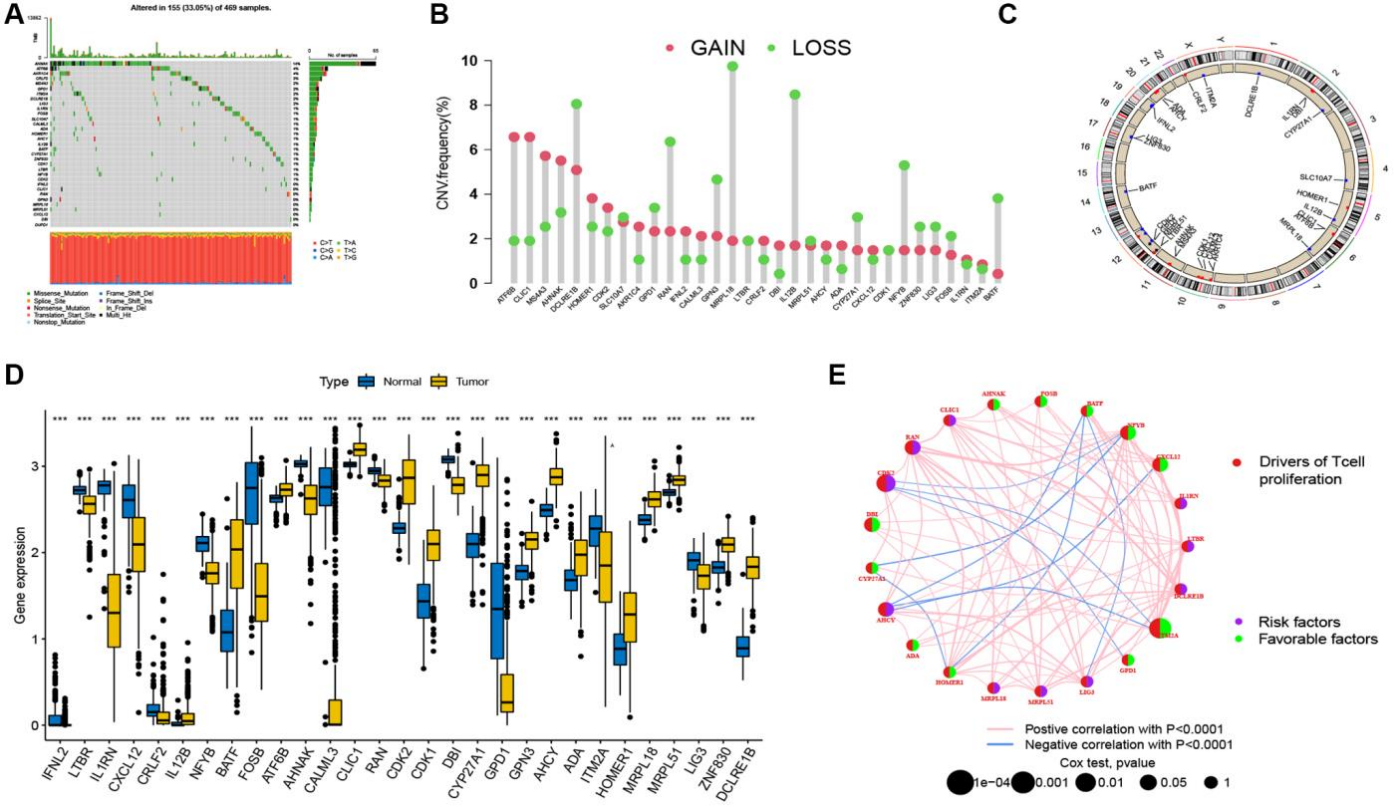
**Genetic and transcriptional analysis of TRGs in SKCM.** (**A**) The incidence of somatic mutations among the 33 TRGs in SKCM patients. (**B**) Frequencies of CNV gain and loss among TRGs. (**C**) Locations of CNV in TRGs on 23 chromosomes. (**D**) Expression levels of differentially expressed TRGs between normal and tumor samples. (**E**) Network of the comprehensive landscape of TRGs interactions in melanoma. The lines connecting the genes represent their interactions. Blue and red represent positive and negative correlations. ^*^p < 0.05; ^**^p < 0.01; ^***^p < 0.001.

Figure 1 illustrates a map of the current work’s process. This study included all 33 TRGs for analysis. To explore the variation of TRGs in melanoma patients, we performed a comprehensive analysis of the somatic mutations in the 33 TRGs, which revealed 155 (33.05%) somatic mutations among the 468 SKCM patients (Figure 2A). Of these, AHNAK had the highest mutation rate (14%), followed by ATF6B, while nine TRGs (IFNL2, CLIC1, RAN, GPN3, MRPL18, MRPL51, CXCL12, DBI and DUPD1) did not have any mutations. Next, the prevalence of CNV in TRGs was evaluated. Among them, there was a general increase in CNV in ATF6B, CLIC1, MS4A3 and AHNAK, while DCLRE1B, RAN, GPN3, MRPL18, IL12B, NFγB and BATF showed a decrease in CNV (Figure 2B). Figure 2C illustrates the locations of the CNV alterations of the TRGs on their respective chromosomes. Our results illustrated multiple TRGs are mutated in SKCM patients.

In addition, SKCM patients were compared with normal controls. The expression levels of mRNA were measured, and 29 significantly differentially expressed TRGs were identified between SKCM patients and controls (Figure 2D), which indicated that the expression of TRGs are different between patients and healthy controls. Meanwhile, the overall situation, including TRG interactions, the connection between influential factors, and their significant prognostic values in SKCM patients are shown in the T cell proliferation network plot (Figure 2E).

### Identification of two TRG molecular clusters in SKCM

**Figure 3:**
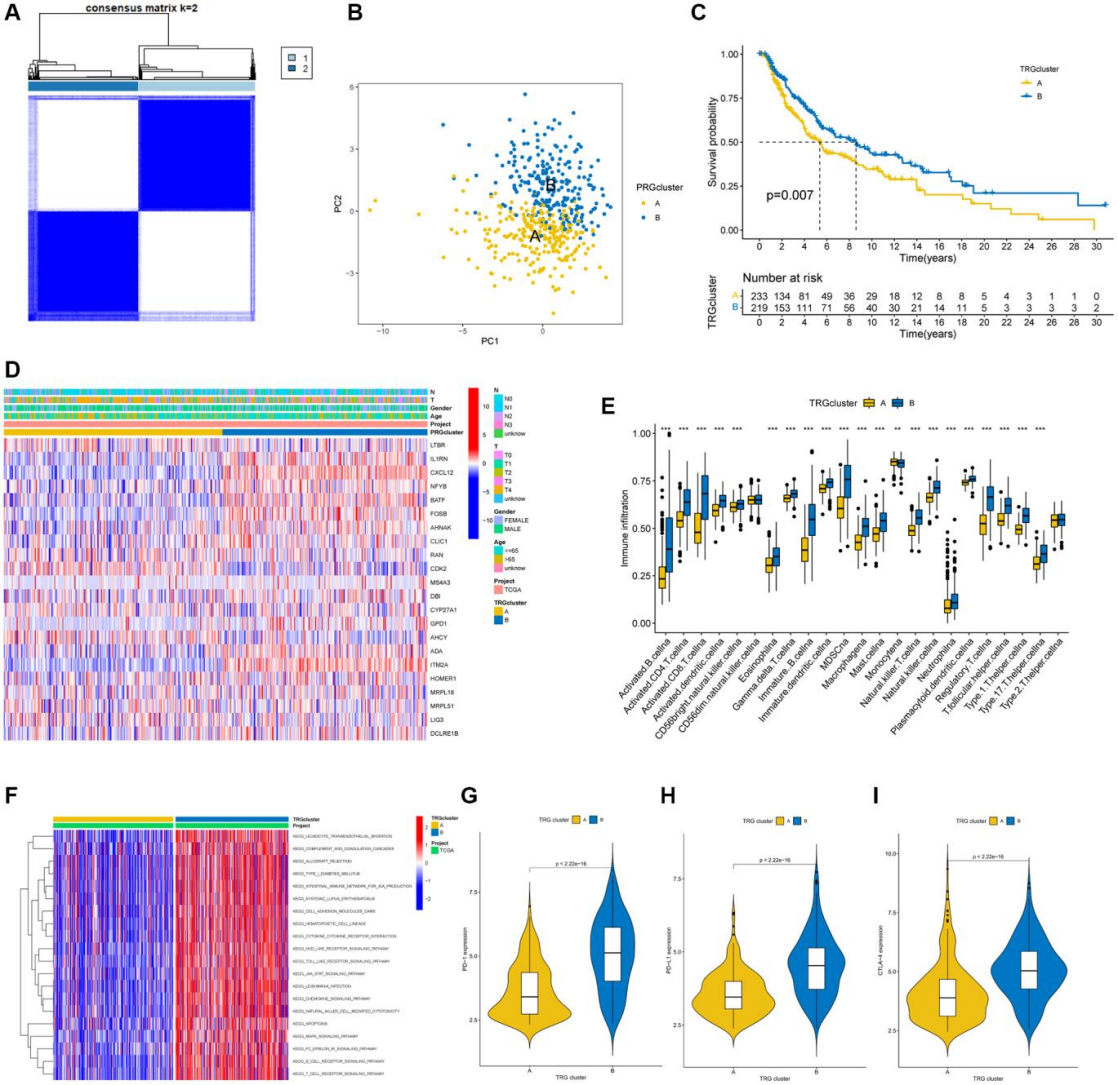
**TRG clusters in melanoma samples and clinical characteristics, tumor microenvironment between two clusters.** (**A**) Two TRG clusters were identified using consensus clustering analysis. (**B**) PCA demonstrated a great difference between the two TRG clusters. (**C**) The K-M curve illustrated the difference in survival time between the two TRG molecular clusters (*p* = 0.007); (**D**) Heatmaps demonstrated the distinctions between TRG clusters in clinical features and TRGs expression in SKCM patients. (**E**) Different immune cell infiltration between the two molecular clusters. (**F**) GSVA showed the enriched pathways in TRG clusters, in which red and blue represent activated and inhibited pathways, respectively. (**G**–**I**) Expression of PD-1, PD-L1, and CTLA-4 in the two TRG clusters. ^**^p < 0.01; ^***^p < 0.001.

To further understand the impact of TRGs on survival in SKCM patients, a consensus clustering algorithm based on 33 TRG expression levels was used to classify all SKCM patients into two TRG molecular clusters (Figure 3A). According to the study results, k = 2 was an effective option for cluster A patient’s classifications (*n* = 241) and cluster B (*n* = 227). Next, a PCA analysis was performed on the two TRG molecular subtype groups, revealing significant differences in the TRG expression conditions (Figure 3B). Based on the KM curves, patients with cluster B had a better survival time than patients with cluster A (*p* = 0.007; Figure 3C). The association between the clinical characteristics, TRG cluster, and TRG expression is shown in a heatmap (Figure 3D). The ssGSEA results revealed higher immune cell infiltration levels in molecular cluster B than in molecular cluster A (Figure 3E). GSVA demonstrated significant enrichment of important biological pathways in subtype B, including leukocyte transendothelial migration, complement-and-coagulation-cascades, etc. (Figure 3F). Furthermore, our results showed higher PD1, PD-L1, and CTLA- 4 genes in subtype B expression levels, (Figure 3G–3I) compared to subtype A.

The above results indicate that the prognosis and some other clinical features of the two molecular clusters are different, and we could find DEGs between the two clusters for further study.

### Identification of two gene clusters based on DEGs in SKCM patients

**Figure 4:**
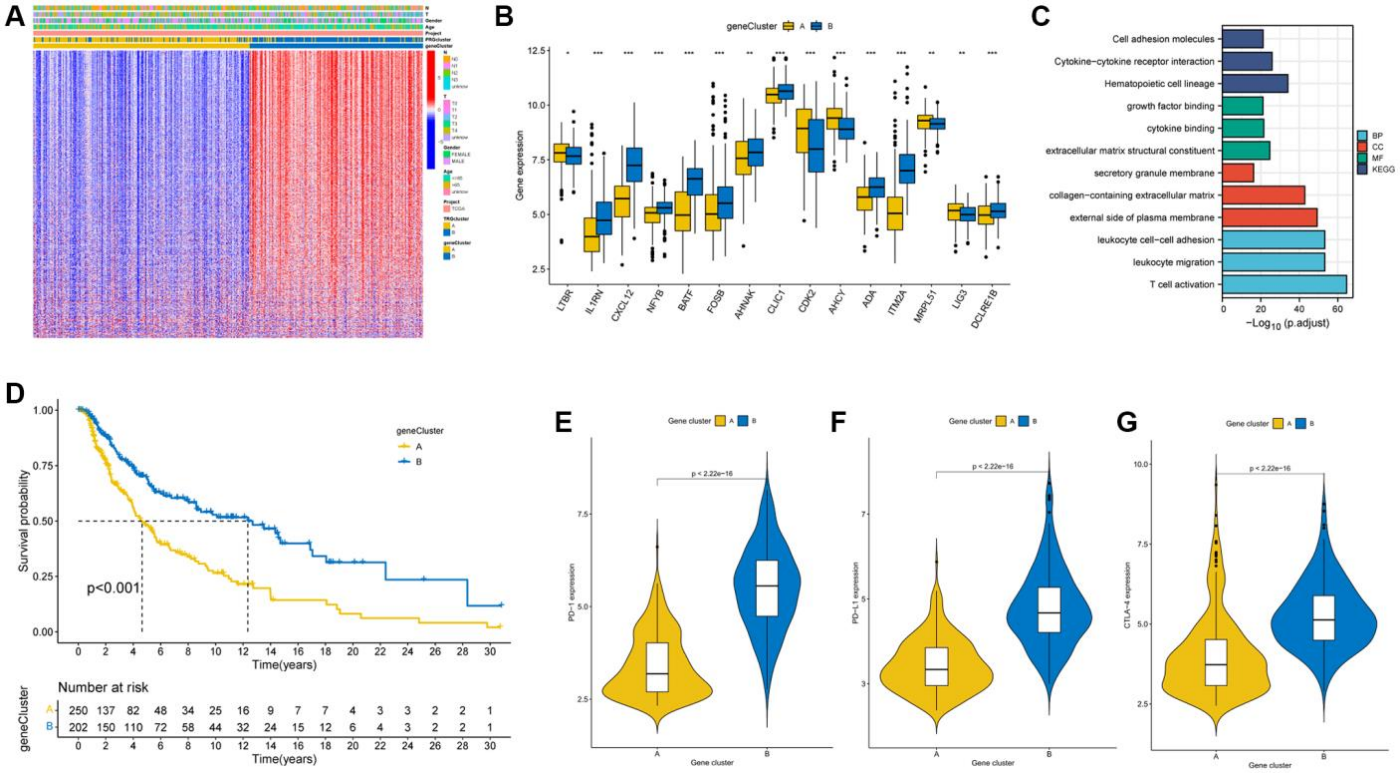
**Identification of gene clusters based on DEGs.** (**A**) Heatmap demonstrated the relation between the two gene clusters and clinical conditions. (**B**) Expression levels of DEGs in the two gene clusters. (**C**) GO and KEGG analysis of DEGs. (**D**) The K-M curve showed higher survival in patients in cluster B. (*p* < 0.001). (**E**–**G**) Expression levels of PD-1, PD-L1, and CTLA-4 in the two gene clusters. ^*^p < 0.05; ^**^p < 0.01; ^***^p < 0.001.

We identified 1249 DEGs based on the two distinct TRG molecular groups. A univariate Cox regression analyses of these 1249 DEGs identified 827 prognostic DEGs (*p* < 0.05) associated with overall survival (OS) for subsequent analyses. Subsequently, a consensus clustering algorithm was applied to split SKCM patients to two gene clusters (cluster A and cluster B) according to the prognostic DEGs. The relationship between clinical characteristics, TRG cluster, gene cluster, and DEGs are presented in Figure 4A. The differences in differentially expressed TRGs between the two distinct gene subtypes are shown in the boxplot (Figure 4B). Moreover, GO, and KEGG analyses were conducted, including significant cellular components (CC), biological processes (BP), molecular functions (MF), and pathways (Figure 4C). These prognostic DEGs are majorly correlated with the BP of leukocyte cell-cell adhesion, leukocyte migration and T-cell activation. The DEGs are associated with the CC of secretory granule membrane, collagen-having ECM, and external side of plasma membrane, which are also involved in the MF of growth factor binding, cytokine binding and ECM structural constituent. These DEGs participate in several pathways, including cell adhesion molecules, cytokine-cytokine receptor interaction and hematopoietic cell lineage.

KM curves revealed better survival for patients with gene cluster B compared to cluster A (log-rank test, *p* < 0.001; Figure 4D). Meanwhile, elevated expression of PD1, PD-L1 and CTLA-4 genes was shown in gene cluster B (Figure 4E–4G).

### Constructing and validating a TRG_score model

**Figure 5:**
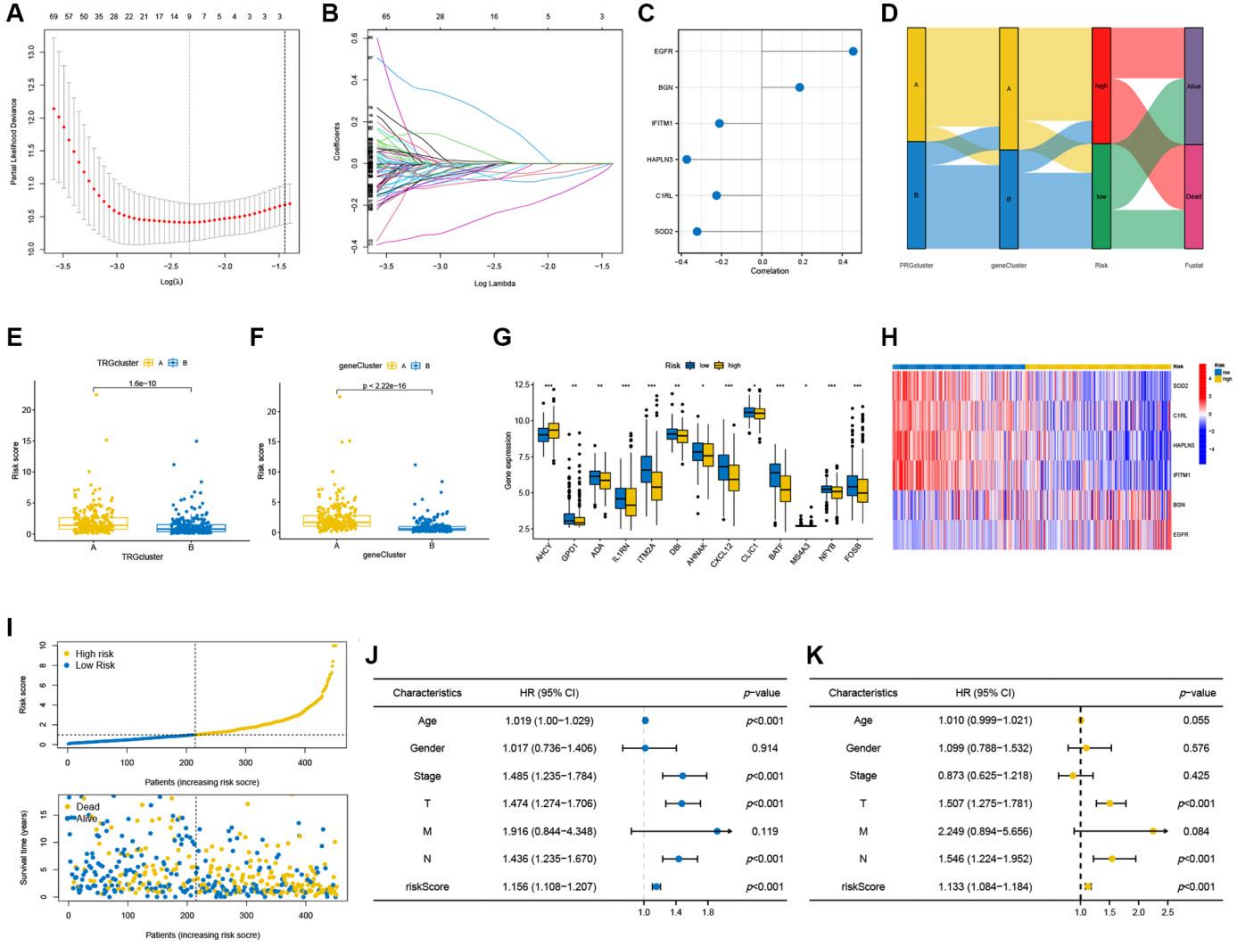
**Construction of the prognostic model.** (**A**, **B**) The LASSO regression analysis and partial likelihood deviance on the prognostic genes. (**C**) Forest plot of the multivariate cox regression analysis for the six signature genes. (**D**) Sankey diagram demonstrated the distribution of TRG subtypes, gene subtypes, risk groups and survival status in SKCM patients. (**E**, **F**) Differences in risk score between the two TRG clusters and two gene clusters. (**G**) Differences in expression levels of TRGs in the two risk groups. (**H**) Heatmap illustrating the expression of six signature genes in the two risk groups in the testing cohort. (**I**) Risk score and survival outcome of each sample. Forest plots of univariate (**J**) and multivariate (**K**) Cox regression analyses in SKCM patients. ^*^p < 0.05; ^**^p < 0.01; ^***^p < 0.001.

With we have confirmed DEGs identified based on the two TRG molecular clusters are associated with patients’ survival, we could calculate risk scores based on these genes. LASSO and multivariate Cox regression analyses were done among all of prognostic DEGs to screen out six prognostic genes (SOD2, C1RL, HAPLN3, IFITM1, BGN and EGFR) that were used to construct the TRG_score model. The processes for the LASSO regression are shown in Figure 5A, 5B, and coefficient values of the multivariate Cox regression are displayed in Figure 5C and Supplementary Table 1. A TRG_score model was constructed utilizing regression analyses results as follows:

Risk score = (−0.3217 × expression of SOD2) + (−0.2248 × expression of C1RL) + (−0.3732 × expression of HAPLN3) + (−0.2107 × expression of IFITM1) + (0.1884 × expression of BGN) + (0.4546 × expression of EGFR).

A Sankey diagram was used to illustrate classification of SKCM patients in the two TRG molecular groups, two gene groups and two risk-score groups (two groups) according to their risk scores (Figure 5D). The risk scores for the two TRG groups (Figure 5E) and the two gene groups (Figure 5F) are depicted in their respective boxplots. Figure 5G illustrates differential expression of TRGs through two risk groups, and expression differences for 6 signature genes are shown in the heatmap (Figure 5H). Patients with SKCM with low-risk scores had better overall survival (Figure 5I). Univariate (Figure 5J, *p* < 0.001) and multivariate (Figure 5K, *p* < 0.001) cox regression analyses were performed, revealing that age, gender, tumor staging, and risk score might serve as independent predictive variable.

**Figure 6:**
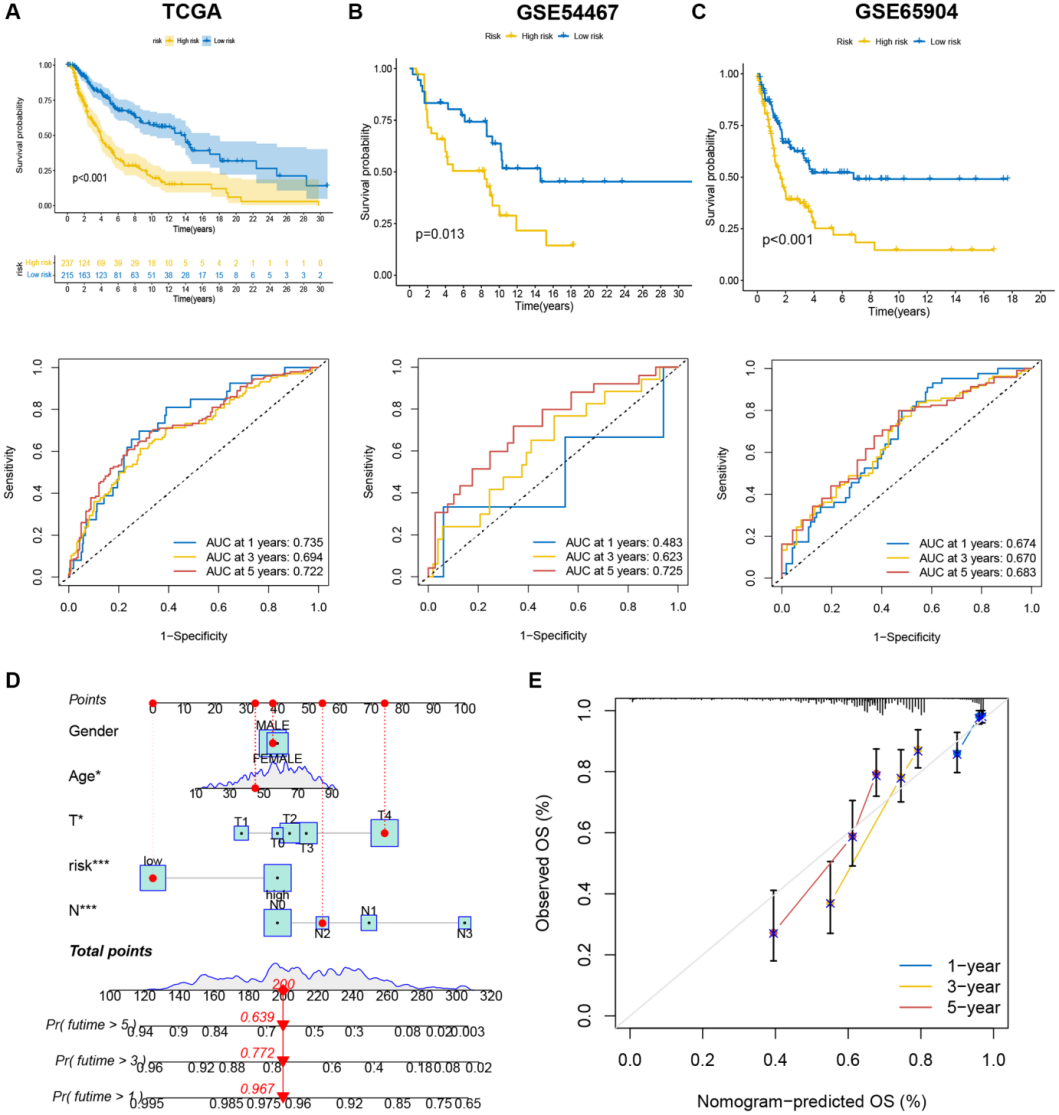
**The efficiency of the risk score and comprehensive score in predicting patient survival.** (**A**–**C**) K-M analysis and ROC curves showed the prognostic value in training and validation cohorts. (**D**) The nomogram showed the prognostic value of clinicopathological parameters and risk score. (**E**) The calibration plots indicate the accuracy and specificity of the nomogram.

The accuracy of risk score for forecasting survival for SKCM patients was assessed using KM curves and area under curves (AUC). The survival analysis results for training cohort from TCGA showed that low-risk patients had more favorable outcomes (*p* < 0.001), with the 1-, 3-, and 5-year AUC values being 0.735, 0.694, and 0.722, respectively (Figure 6A). The results for two validation cohorts, GSE54467 (Figure 6B, *p* = 0.013, 1-year AUC = 0.483, 3-year AUC = 0.623, 5-year AUC = 0.725) and GSE65904 (Figure 6C, *p* < 0.001, 1-year AUC = 0.674, 3-year AUC = 0.670, 5-year AUC = 0.683), revealed that a low risk score was associated with significantly prolonged survival than a high-risk score. These results illustrated that SKCM patients’ survival could be predicted using the risk score. Meanwhile, a nomogram was constructed by integrating risk score and clinical information, as age, gender and tumor stage, to predict 1-, 3- and 5-year survival time (Figure 6D). The calibration curve showed that nomogram model predicted survival well based on the closeness of anticipated and observed OS values (Figure 6E).

### Evaluation of TME, TMB and CSC index among high- and low-risk groups

**Figure 7:**
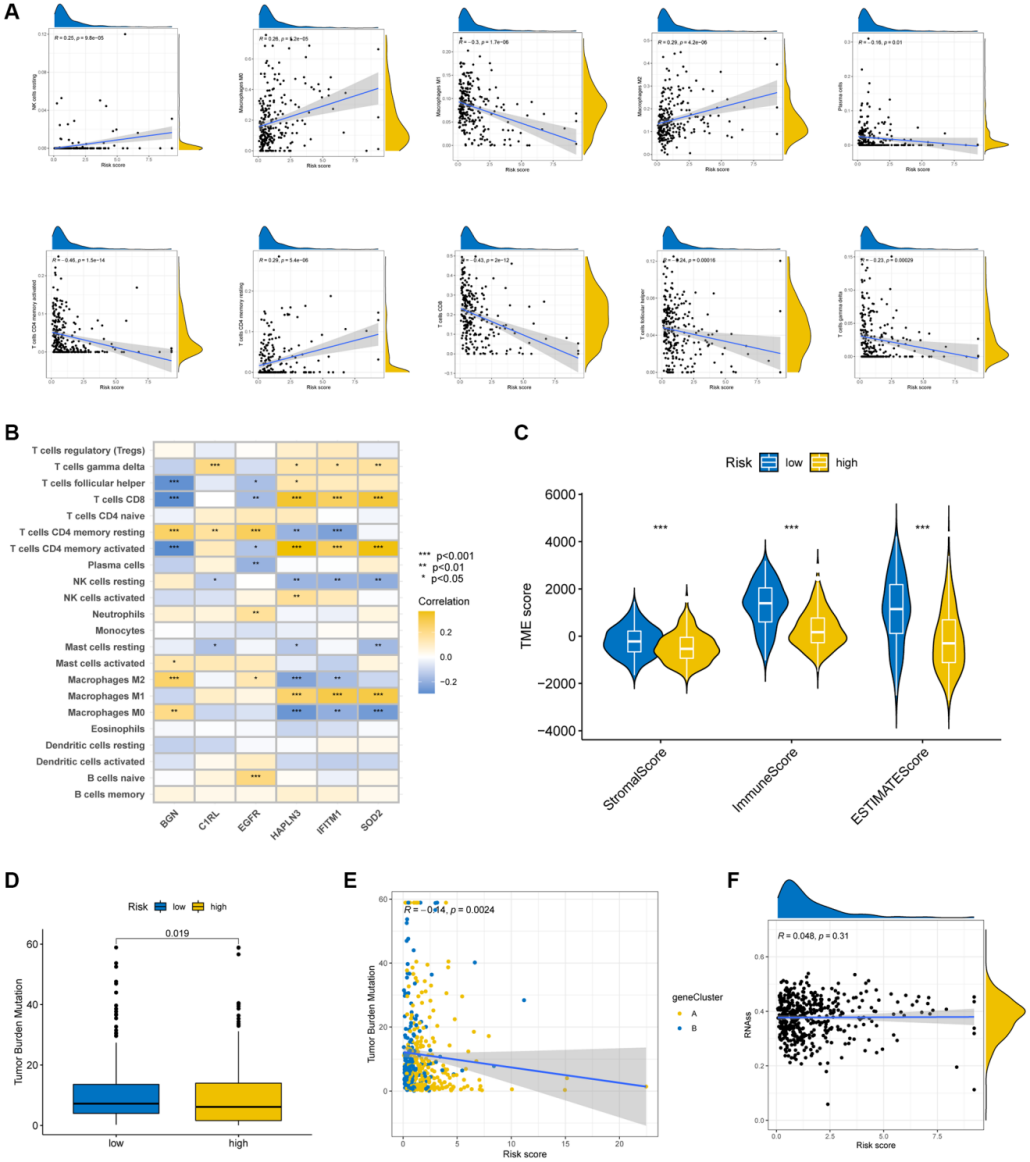
**Evaluation of TME in the high-risk and low-risk groups.** (**A**) Relationship between risk score and a series of immune cell types. (**B**) Correlation between the abundance of immune cells and the six prognostic TRGs. (**C**) Correlation between risk score and TME scores. TMB (**D**, **E**) and CSC (**F**) in high- and low-risk groups. ^*^p < 0.05; ^**^p < 0.01; and ^***^p < 0.001.

After identifying the high- and low- risk groups, we also performed some bioinformatics analysis to verify the accuracy of our risk scores. The correlation between risk scores and infiltrate immunological cells was shown in some scatter plots (Figure 7A) which demonstrated the risk score was negatively correlated with M1 macrophages, activated memory CD4 + T cells and CD8 + T cells. The association between the six prognostic genes and immune cell abundance is exhibited in Figure 7B. Significant associations were found between most immune cells and the six signature genes. Moreover, there was a strong correlation across a low-risk score and a high immunological score. (Figure 7C).

In the analysis of mutation data of SKCM patients, the low-risk group had a greater TMB than high-risk group. (Figure 7D), suggesting that low-risk patients may gain from immunotherapies. The Spearman correlation analysis revealed that risk score was inversely correlated with TMB (*p* = 0.002; Figure 7E).

Figure 7F showed no significant association among risk score and CSC index (R = 0.048, *p* = 0.31).

### Immune checkpoint genes expression and IPS between the high-risk group and low-risk group

**Figure 8:**
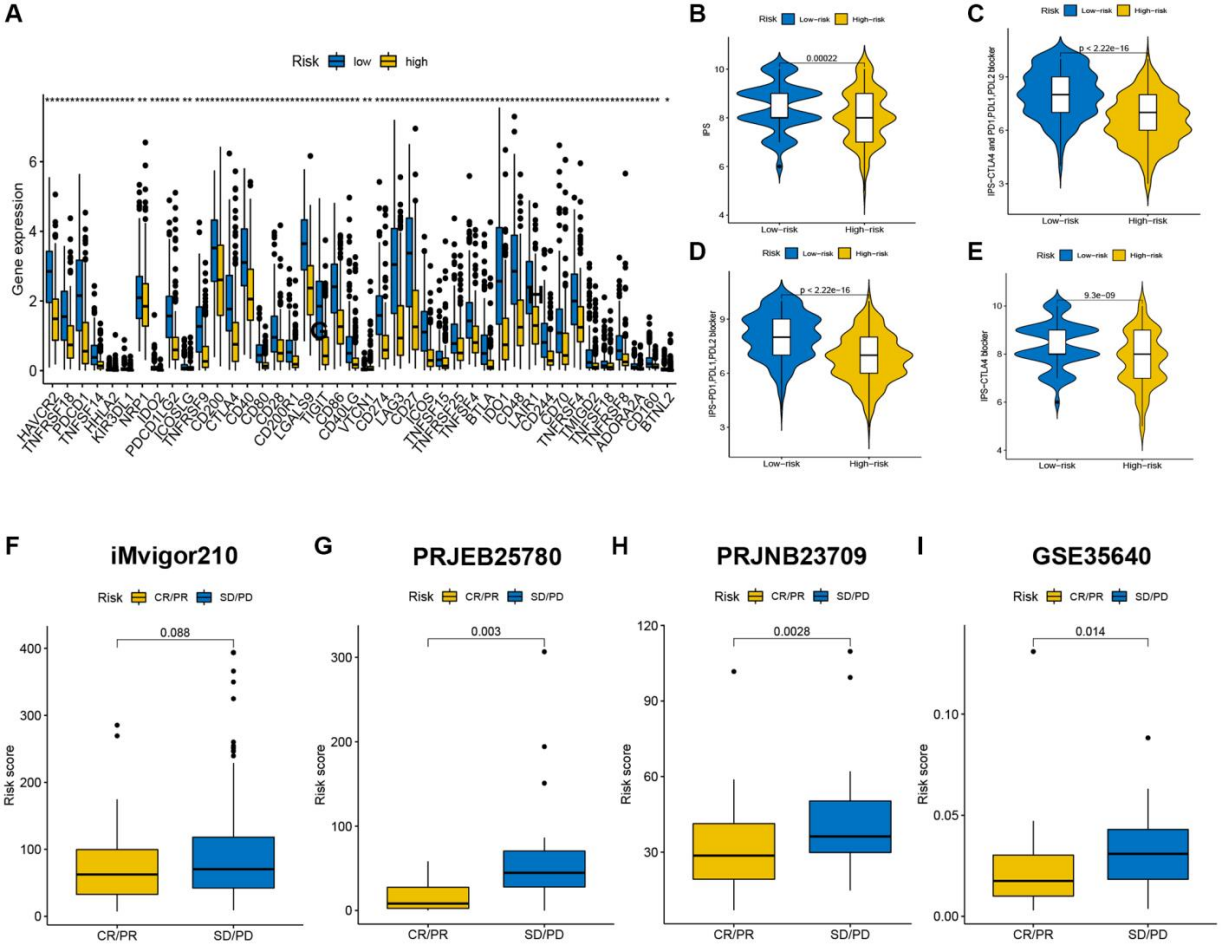
**Immune checkpoint genes expression, IPSs and immunotherapy benefits of patients in the two risk groups.** (**A**) The differences in immune checkpoint gene expression between the high-risk and low-risk groups. (**B**–**E**) CR/PR patients had significantly lower risk scores than SD/PD patients in the iMvigor210 (**F**), PRJEB25780 (**G**), PRJNB23709 (**H**), and GSE35640 (**I**) cohorts.

We further researched expression of immunological checkpoint genes and found higher expression levels in PD-1 (PDCD1), PD-L1 (CD274) and CTLA-4 in low-risk group (Figure 8A; *p* < 0.05), illustrating that SKCM patients with low-risk score may benefit from ICI therapy. Meanwhile, the IPSs of two different risk groups were compared to explore the response of SKCM patients to the ICI blockade therapies. The IPSs of low-risk SKCM patients were significantly higher than in high-risk group (Figure 8B; *p* = 0.00022). Moreover, low-risk patients who received CTLA-4 and PD-1/PD-L1/PD-L2, PD-1/PD-L1/PD-L2 or CTLA-4 blocker therapy had higher IPSs (Figure 8C–8E, *p* < 0.001), indicating a better response to ICI therapy. We also validated efficacy of risk score at forecasting ICI responses in the iMvigor210 (urothelial cancer), PRJEB25780 (metastatic gastric cancer), PRJNB23709 (melanoma), and GSE35640 (melanoma) cohorts. CR/PR Patients were more likely to have a lower risk score, whereas high-risk patients had greater SD/PD (Figure 8F–8I, *p* < 0.05).

### Drug susceptibility analysis

**Figure 9:**
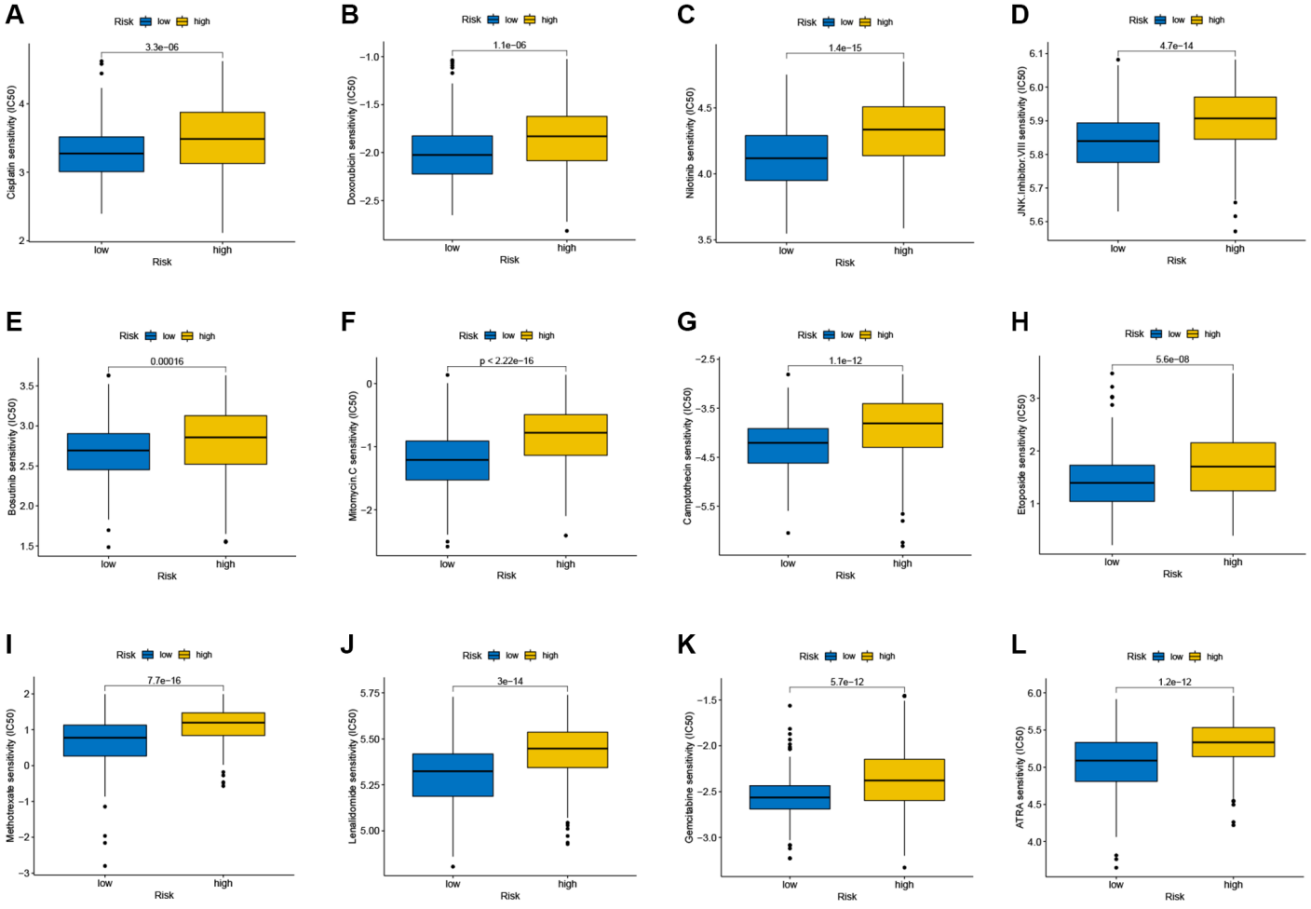
(**A**–**L**) Therapeutic drugs showed significant differences in IC50 between the high- and low-risk groups.

The susceptibility of tumors to some chemotherapy drugs was compared between 2 groups. The results revealed that high-risk SKCM patients had greater IC50 values for cisplatin, doxorubicin, nilotinib and so on, suggesting that TRG is associated with drug susceptibility (Figure 9A–9L).

### Comparison of the expression levels of the six prognostic signature genes between SKCM cells and normal melanocytes

**Figure 10:**
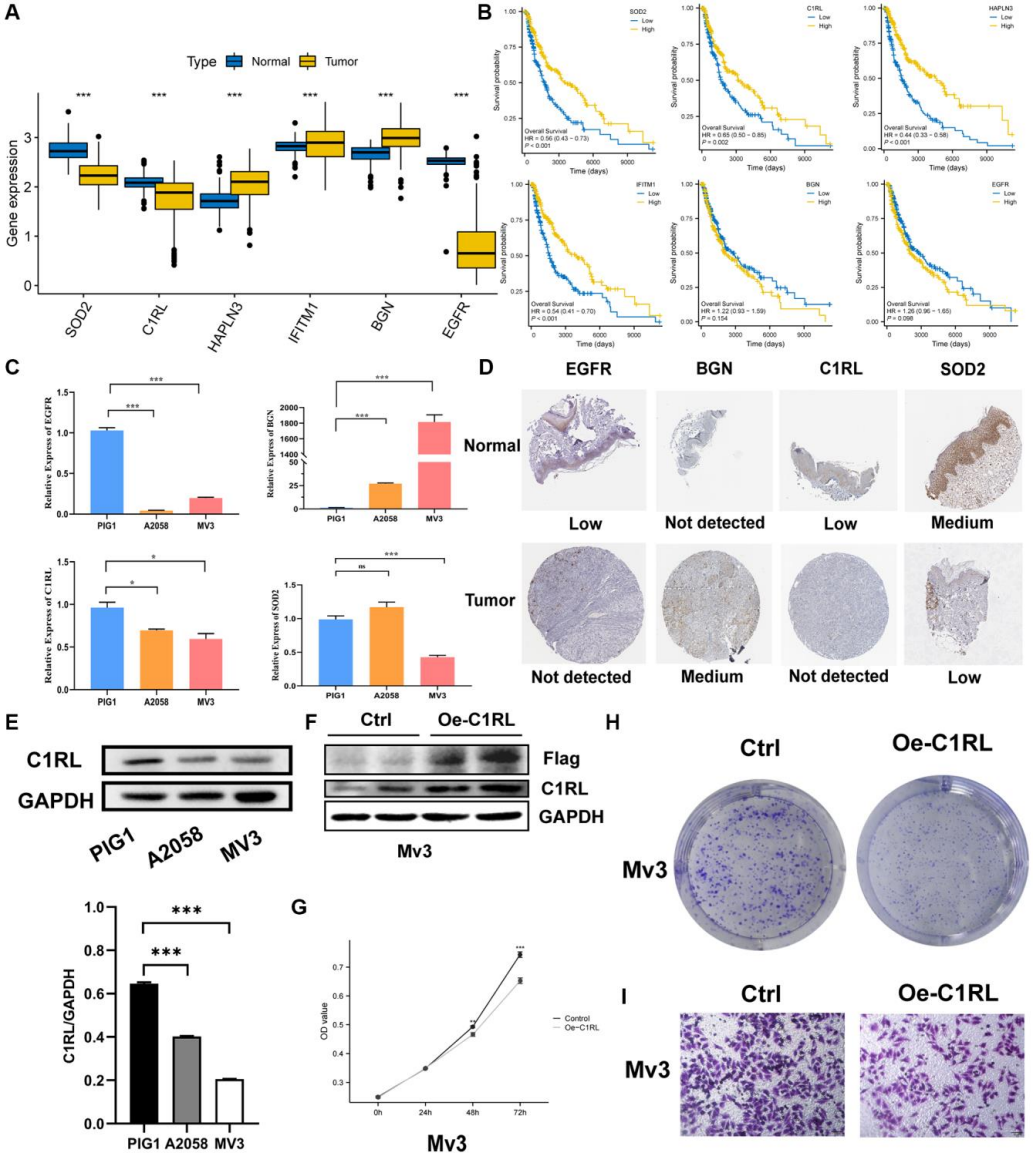
**Prognostic TRGs gene expression levels in tumor and normal cells and functional analysis.** (**A**) Different expressions of the six signature genes between the normal and tumor tissues (TCGA AND GTEx). (**B**) Confirmation of prognostic value of 6 TRGs for patients in TCGA by Kaplan–Meier analysis. (**C**) The relative RNA levels of EGFR, BGN, C1RL AND SOD2 in normal skin melanocyte and melanoma cell lines by q-PCR. (**D**) The IHC staining showed 4 signature genes expression at the protein level. (**E**) The protein level expression of C1RL genes based on melanocytes (PIG1) and melanoma cells lines (A2058 and MV3) by Western blot. (**F**) Western blot is used to assess the Overexpression efficiency of in MV3. CCK8 (**G**) and colony formation assay (**H**) are performed to assess effects of C1RL Overexpression on proliferation of MV3. (**I**) Transwell assay is utilized to evaluate the migration.

In order to validate the reliability of six signature genes, we performed a series of *in vitro* experiments. Different expression levels of the six signature genes were identified between SKCM patients and normal control samples and are depicted in a boxplot (Figure 10A). The relative expression levels of six signature genes were measured in SKCM cells and normal melanocytic nevi cells by qRT-PCR. Our findings in Figure 10C demonstrated significantly different expression levels of four signature genes (EGFR, BGN, C1RL and SOD2) in tumor cells compared to the corresponding normal melanocytes. Furthermore, the difference in protein expression of the four signature genes between SKCM and non-tumor tissues was explored using IHC (immune histochemistry) from the public HPA database. Our previous results were supported by differential expression levels of these four genes among normal skin and SKCM tissues (Figure 10D).

### Overexpression of C1RL inhibits cell proliferation and migration

Patients with high C1RL expression have longer Overall Survival (OS) time tested by Kaplan-Meier (KM) test (Figure 10B). We further investigate the protein level of C1RL in melanoma cell lines. Results from the Western blot assay shown protein level of C1RL were decreased in A2058 and MV3 cells compared with melanocyte PIG1 cells. The protein band were analyzed using Image J, and GAPDH protein was used as an internal control (Figure 10E). To explore the biological function of C1RL in SKCM, we used the lipo 3000 to add C1RL expression in MV3 cell lines. Western blot results presented that C1RL expression levels in overexpression cell lines was significantly rose compared to the control cells (Figure 10F). CCK8 assay presented that overexpression of C1RL inhibited cell proliferation (Figure 10G). Moreover, *in vitro* overexpression of C1RL suppressed the formation of clones (Figure 10H). Furthermore, MV3 cells migration capabilities were also impaired after C1RL Overexpression (Figure 10I).

## DISCUSSION

SKCM, derived from melanocytes, is highly invasive, with early metastasis and poor prognosis [2]. Traditional surgical treatment and gradually emerging immunotherapy are the main treatment methods for melanoma, but they have certain limitations, and it is difficult to significantly improve the survival of patients. Melanoma can occur in all parts of the skin and mucosa, and metastasis is extremely fast, which brings great challenges to surgeons in surgery and postoperative reconstruction [4]. The mechanisms of proliferation, recurrence, metastasis and immune escape of malignant melanoma are very complex. Drug therapy often only works for one mechanism, and even combination therapy cannot completely block all pathogenic mechanisms of tumors [21]. Therefore, in this study, we explored the factors affecting the prognosis of the disease, aiming to find more potential treatments to improve the prognosis of melanoma patients based on these factors. We also established a prognostic model to forecast the prognosis of SKCM patients and assess patient immunotherapy response.

T cells are critical in the immunotherapy of tumors. Several genetic engineering methods are used to increase the recognition of tumor cell antigens [22]. Clinicians usually use gene-engineered T cells to treat tumors to control the cell population [23]. However, most studies have only focused on the mechanisms by which T cells eliminate tumors or on a single type of immune-associated cell [24, 25], which does not clarify the combined function of several TRGs. This study assessed the overall changes in TRGs on levels of transcription and genetics in SKCM patients. All of SKCM patients from TCGA database were categorized into two molecular clusters based on 33 TRGs expressions and we found the expression of TRGs had an impact on SKCM patients’ survival. A significant difference in the TME characteristics was observed between two TRG molecular clusters, revealing a link between TRGs and TME. In addition to TME, virtually every subset of immune cells played a role in cancer biology [26]. At the same time, the results of a functional enrichment analysis performed in the two TRG clusters and their activity suggested that the transcriptome differences in TRGs were significantly correlated with immune-associated biological pathways. Moreover, SKCM patients were divided into two gene clusters according to the DEGs between two TRG molecular clusters and we found there is a significant difference in survival between two gene subtypes. According to these findings, DEGs based on the TRG clusters could be used to forecast SKCM patients’ clinical outcomes. Subsequently, a valid prognostic TRG_score model was constructed and showed its predictive power. The signature model included six genes, and biological experiments such as qRT-PCR were carried out to investigate the expression levels between tumor and non-tumor cells. Our results showed the different expression levels of four signature genes across tumor and non-tumor cells, which indicated the reliability of the signature genes. Patients with distinct risk scores had significantly different clinical features, mutations, prognosis, TME, TMB, immunological checkpoint genes expression, IPS and drug sensitivity. Additionally, we built a nomogram based on the TRG_score model by integrating the risk score with clinical characteristics. This model can be used to classify SKCM patients into two distinct risk groups, which may predict prognosis of SKCM patients and deliver novel approaches for immunotherapies.

Although SKCM is generally considered a cancerous malignancy without many therapeutic options, innovative therapies targeting susceptible genes and immune checkpoints have greatly improved patients’ outcomes due to improved biologic understanding and unprecedented innovations [27]. Over the past few decades, ICI therapy has resulted in a significant increase in 5-year survival rate of about <5% to 30% for patients with melanoma [28–30]. ICIs target the dysfunctional immune system and induce CD8-positive T cells to kill tumor cells [31]. Current therapies have revolutionized the standard of care for SKCM patients, but low response rates and inevitable treatment resistance may prevent further improvement in treatment outcomes [12]. Perhaps our research can shed more light on the treatment of melanoma. In our research, we explored the expression of several major immunological checkpoint genes among the high- and low-risk group of patients. The findings revealed that the low-risk patients had elevated expression of immunological checkpoint genes and better survival, indicating that patients with low-risk scores may benefit from ICI therapy. Thus, we concluded that expression levels of immunological checkpoint genes could be used as an indicator to assess effect of immunotherapy in SKCM patients. Furthermore, high tumor mutation burden (TMB-H) is a major candidate biomarker in immune checkpoint inhibitor therapy to identify tumor patients that may benefit from therapy. The underlying assumption is that increasing the number of mutant proteins will generate antigenic peptides that may enhance immunogenicity [32–35]. This study showed low-risk patients exhibited a high TMB and a good prognosis. These results demonstrated accuracy of our prognostic model in assessing patient risk from another perspective.

The TME is consisting of stromal fibroblasts, infiltrating immune cells, blood vessels, lymphatics, and a noncellular component, such as the extracellular matrix (ECM) [36]. The leading theory is that the immune system is majorly responsible for eliminating a large proportion of nascent tumor cells. However, cancer progression may be supported by immune and inflammatory cells infiltrating the tumor [37]. All immunological cell types can be shown in TME, as macrophages, naive and memory lymphocytes B cells, effector T helper (Th) cells and so on [38]. Several growth factors and cytokines are released by these immune and inflammatory cells, as well as enzymes that degrade the extracellular matrix, which could promote tumor development and growth [38]. All of these risk factors could influence the survival of melanoma patients [39]. In addition, the high-risk SKCM patients showed more infiltrating immune cells in TME and worse prognosis, further suggesting that immune cells located in TME could accelerate tumor progression and influence tumor prognosis. The results also validated the efficiency of our signature model in evaluating patient risk.

At present, effective non-invasive treatment for melanoma is limited in clinical practice [40], so we performed a drug-sensitivity analysis. The results revealed that low-risk score group was more sensitive to a variety of chemotherapeutic drugs, such as cisplatin, doxorubicin, nilotinib and so on, which might open more options for the selection of therapeutic drugs for SKCM. Notably, the expression levels of six signature genes were evaluated in two SKCM cell lines and one normal melanocyte line, revealing different expression levels of SOD2, BGN, EGFR and C1RL between SKCM and normal cells. The results implied that the four genes might be new therapeutic targets for melanoma. Complement C1r subcomponent like (C1RL) has been found to be a prognostic marker in a variety of tumors [41, 42], but whether it plays a role in SKCM has not been elucidated. In our experiments *in vitro*, we found the melanoma cell lines of C1RL overexpression grew more slowly and reduced migratory capacity. The results implied that the four genes, especially C1RL, may have a significant function in melanoma and may be new therapeutic targets for melanoma.

However, the limitations of the current study should be acknowledged. This was a retrospective study, and all analyses were performed on a public database. Thus, our findings need to be confirmed by more experiments *in vivo* or *in vitro* to improve our understanding of the mechanism of SKCM development and the role of TRGs. Moreover, some key clinical features, such as surgery and responses to chemotherapy, were not available in the databases, which may impact the accuracy of some clinical studies.

## MATERIALS AND METHODS

### Data collection

The gene expression data (fragments per kilobase million, FPKM), genetic mutation information, and clinical manifestation data of SKCM patients were downloaded from The Cancer Genome Atlas (TCGA) database (Supplementary Table 2), while the information of normal control groups was obtained from the Genotype-Tissue Expression (GTEx) database. TCGA database values were converted to transcripts per kilobase million (TPM) utilizing R studio software (version1.4.1106). The dataset from the TCGA database included 471 melanoma samples and 1 normal sample. Cases with incomplete clinical data were excluded, and the 468 remaining tumor samples were retained for subsequent analyses. The two SKCM datasets, GSE54467 and GSE65904 (Supplementary Table 3), were obtained from a publicly available database, the Gene Expression Omnibus (GEO), and were used for verification. Additionally, four groups of immunotherapy-associated data (iMvigor210, PRJEB25780, PRJNB23709 and GSE35640) were obtained from the website http://research-pub.gene.com/IMvigor210CoreBiologies, TIDE website (http://tide.dfci.harvard.edu/) and GEO (Supplementary Table 4). These datasets included urothelial, metastatic gastric cancers and melanoma and compared the effects of immunotherapy with programmed cell death-1(PD-1), programmed cell death-1 ligand 1 (PD-L1) or cytotoxic T-lymphocyte-associated protein 4 (CTLA-4) blockade therapy, which were used to assess the efficiency of our prognostic model in forecasting immunotherapy outcomes.

### Genetic and transcriptional analysis of TRGs in SKCM

Thirty-three TRGs were identified in a recent study. These gene names and their full details of expression condition were listed, and the somatic mutations were represented by generating a waterfall plot with the maftools R package. Transcriptional mutation data of the 33 TRGs (Supplementary Table 5) were retrieved from the TCGA database for analyzing the copy number variation (CNV) frequency and corresponding location information. Using the limma R package, the Wilcoxon signed-rank test was used to compare TRG expression between normal and tumor tissues. The *p*-value of survival analysis for TRGs was calculated using the Log-rank test, and the interactions among the TRGs were also explored using correlation analysis.

### Clustering analysis for TRG molecular subtypes

ConsensusClusterPlus R package was utilized to split all SKCM patients into two TRG molecular clusters depending on TRGs expression. K-means algorithm was used to identify optimal subtype numbers. Then, the limma and ggplot R packages were employed to establish principal component analysis (PCA) in order to distinguish the two identified TRG molecular subtypes.

### Analysis of the clinical and biological features of the two TRG molecular clusters

To observe the prognostic condition of the two TRG molecular clusters, a survival analysis was done using survival and survminer R packages. Kaplan-Meier (KM) curves were obtained to evaluate differences in survival time between the two subtypes.

Patients’ age, sex, TNM stage and TRGs expression information were visualized by a heatmap generated by using the pheatmap R package. Then, the difference in immunological cell infiltration among two molecular groups was assessed by gene set variation analysis (GSVA) utilizing GSVA R. Moreover, single-sample gene set enrichment analysis (ssGSEA) was served to explore immune-related pathways in the different TRG clusters, and results were visualized with pheatmap packages.

Based on the Wilcoxon signed-rank test, PD-1, PD-L1, and CTLA-4 levels in two different TRG clusters were compared and described using violin plots.

### DEGs analysis and enrichment analysis

The DEGs across distinct TRG molecular clusters were detected utilizing limma R with a |log FC| >1 and an adjusted *p*-value < 0.05. To investigate prospective roles of DEGs, Gene Ontology (GO) and Kyoto Encyclopedia of Genes and Genomes (KEGG) analyses were done. Furthermore, ConsensusClusterPlus R package was utilized to classify all SKCM patients into two gene clusters regarding DEGs expression, and survival analysis was performed to assess distinct survival among two gene subtypes. Additionally, expression conditions of three major immunological checkpoint genes, PD-1, PD-L1, and CTLA-4, were compared among two gene clusters.

### Construction and evaluation of the prognostic TRG_score model

TRG risk score was obtained to evaluate risk of each cancer patient, and a prognostic TRG _score model was established. First, Lasso and multiple Cox regression analyses and cross-validation were performed on TRGs using the glmnet R package to find signature genes that predict the prognosis of SKCM patients. A prognostic TRG score model was developed based on these signature genes.

TRG_score was assessed as follows:


$$\text{TRG\_score}=\text{Σ }\left(\text{Expi}\times \text{coefi}\right)$$

Where Expi and Coefi indicate expression of each gene and risk coefficient, respectively.

According to their risk assessments, patients were split into high- and low-risk groups. The relationships between risk score and TRG clusters, and gene clusters were analyzed. Furthermore, risk score univariate and multivariate Cox regression analysis and some clinical features to identify independent prognostic factors in SKCM patients. The efficiency of TRG _score model in forecasting SKCM patients’ survival was validated in two independent cohorts (GSE54467 and GSE65904) using the survival analysis and receiver operating characteristic (ROC) methods. A characteristic nomogram based on sex, age, tumor staging system, and risk score was then constructed to forecast 1-, 3- and 5-year survival for each SKCM patient. The calibration plots of nomogram were then developed by survival and rms R package.

### Immune status, TME, TMB and cancer stem cell (CSC) index differences between the high-risk group and low-risk group

Common chemotherapy medications (paclitaxel, doxorubicin, bexarotene, bicalutamide, imatinib, and tiifarnib) were evaluated utilizing half-maximal inhibitory concentration (IC50) and R package “pRRophetic” utilizing info from Genomics of Drug Sensitivity for Cancer.

### Differences in immune checkpoint gene expression and immunological cell proportion score (IPS) between the high- and low-risk groups

Wilcoxon signed-rank test was utilized to compare expression of immunological checkpoint genes among 2 groups. Different types of ICI treatments, as PD-1/PD-L1/PD-L2/CTLA-4, PD-1/PD-L1/PD-L2 and CTLA4 blockers, were predicted by IPS in patients. Furthermore, complete response (CR)/partial response (PR) and stable disease (SD)/progressive disease (PD) values were calculated using the iMvigor210, PRJEB25780, PRJNB23709 and GSE35640 cohorts.

### Drug susceptibility analysis

pRRophetic R assessed IC50 values of major chemotherapy agents to explore differences in their therapeutic effects among the two groups.

### The expression condition analyses of the six prognostic TRGs between normal and SKCM cells

The transcriptional levels of the six prognostic TRGs of the SKCM and normal groups were compared. qRT-PCR was performed on one normal melanocyte line (PIG1) and two SKCM cell lines (A2058, MV3) to compare the expression levels of the six prognostic signature genes. Furthermore, the expression conditions of the key TRGs between SKCM and normal groups were observed based on the Human Protein Atlas (HPA) database.

### Cell culture and *in vitro* validation via qRT-PCR and Western blotting

The normal human skin melanocyte cell line (PIG1) and human melanoma cell lines MV3 and A2058 were bought from American Typical Culture Center (ATCC). All cell lines were cultured in DMEM (HyClone) with 10% FBS (Lonsera) and 1% double antibody (streptomycin and penicillin) at a temperature of 37°C and 5% CO2.

Gene expression levels were determined using qRT-PCR. TRIzol kit (Carlsbad, CA, USA) was utilized to isolate RNA from A2058, MV3 and PIG1 cell lines, and the RNA concentration was determined by ultraviolet spectrophotometry. Then, PrimerScript RT Master reverse-transcribed RNAs into DNA (cDNA). The concentration of cDNA was measured utilizing SYBR Green PCR master mix and the LightCycler 96 System (Roche). The relative mRNA expression levels of EGFR, BGN, SOD2, C1RL, HAPLN3 and IFITM1 were assessed by 2−ΔΔCt assay and normalized by 36B4, which was used as the internal reference. *T*-tests were utilized to compare expression for various cell lines (Supplementary Table 6).

Total proteins were extracted from tissues and cells using RIPA buffer (Beyotime, China) supplemented with protease inhibitor and phosphatase inhibitor. Western blotting was performed according to the protocol as described previously [43]. The primary antibodies used in the study were anti-GAPDH (Proteintech, China), anti-C1RL (Zenbio, China).

### Cell transfection

For transfection of plasmids (PCDH-GFP+PURO-3xFlag or C1RL PCDH-GFP+PURO-3xFlag, Youbao, Wuhan, China), cells were grown to 70% confluence and were transfected using Lipofectamine 3000 (Invitrogen, Shanghai, China) based on the protocol of the manufacturer. After incubating for 72 hours, cells were washed and used for subsequent experiments.

### Cell proliferation and migration assay

MV3 cells (200000/well) were cultured in 6-well plates and transfected with PCDH-GFP+PURO-3xFlag or C1RL PCDH-GFP+PURO-3xFlag. 72 hours after transfection, 1500 cells were arranged into 96-well plates. After culture for 0, 24, 48, or 72 h with PCDH-GFP+PURO-3xFlag or C1RL PCDH-GFP+PURO-3xFlag, cells were cultured with the CCK8 solution (C0038, Beyotime, Shanghai, China) for an additional 1.5 h. An optical density (OD) value at 450 nm was used to evaluate cell viability.

In order to examine the effects of C1RL expression on human melanoma cell proliferation, the above transfected MV3 cells (1500/well) transfected with PCDH-GFP+PURO-3xFlag or C1RL PCDH-GFP+PURO-3xFlag were added to the 6-well plates. After ten days, the number of colonies were counted.

Transwell chambers (Corning, NY, USA) were used for migration experiment. The above transfected MV3 Cells (3 × 10^4^) were suspended in 200 μl serum-free medium and positioned in the top chamber when a medium containing 10% fetal bovine serum was used in the bottom chambers. After incubation for 36 h, inner chambers were scrubbed and cells at the other side of the membrane were exposed 4% formaldehyde solution to fixing, staining with crystal violet and recorded under a microscope.

## Supplementary Materials

Supplementary Tables 1, 5 and 6

Supplementary Tables 2-4
