# Supplementary material for: Development and validation of a novel T cell proliferation-related prognostic model for predicting survival and immunotherapy benefits in melanoma
Source: Aging (Albany NY). 2023 May 24;15(10):4444–64. doi: 10.18632/aging.204748 (PMC10258015; doi:10.18632/aging.204748)
Supplement: Supplementary Tables 1, 5 and 6 [file aging-15-204748-s001.pdf]

SUPPLEMENTARY TABLES

Supplementary Table 1. Coefficient values of 6 signature genes in the multivariate Cox analysis.

Id	Coef
SOD2	−0.321664227
C1RL	−0.224770485
HAPLN3	−0.373204795
IFITM1	−0.210704663
BGN	0.188468569
EGFR	0.454558562

Supplementary Table 5. Summary of 33 recognized drivers of T cell proliferation-related genes.

Gene	Type
IFNL2	drivers of T cell proliferation
LTBR	drivers of T cell proliferation
IL1RN	drivers of T cell proliferation
CXCL12	drivers of T cell proliferation
CRLF2	drivers of T cell proliferation
IL12B	drivers of T cell proliferation
NFYB	drivers of T cell proliferation
BATF	drivers of T cell proliferation
FOSB	drivers of T cell proliferation
ATF6B	drivers of T cell proliferation
AHNAK	drivers of T cell proliferation
SLC10A7	drivers of T cell proliferation
CALML3	drivers of T cell proliferation
CLIC1	drivers of T cell proliferation
RAN	drivers of T cell proliferation
CDK2	drivers of T cell proliferation
MS4A3	drivers of T cell proliferation
CDK1	drivers of T cell proliferation
DBI	drivers of T cell proliferation
CYP27A1	drivers of T cell proliferation
AKR1C4	drivers of T cell proliferation
DUPD1	drivers of T cell proliferation
GPD1	drivers of T cell proliferation
GPN3	drivers of T cell proliferation
AHCY	drivers of T cell proliferation
ADA	drivers of T cell proliferation
ITM2A	drivers of T cell proliferation
HOMER1	drivers of T cell proliferation
MRPL18	drivers of T cell proliferation
MRPL51	drivers of T cell proliferation
LIG3	drivers of T cell proliferation
ZNF830	drivers of T cell proliferation
DCLRE1B	drivers of T cell proliferation

Supplementary Table 6. The primer sequences for qRT-PCR.

Gene	Primer sequence
SOD2	F: 5′-GCTCCGGTTTTGGGGTATCTG-3′<br>R: 5′-GCGTTGATGTGAGGTTCCAG-3′
C1RL	F: 5′-TACCCAGAGCCGTATGGCAA-3′<br>R: 5′-GAACCGACGAATGAGATTGTGA-3′
HAPLN3	F: 5′-CCAGACAGGACTCCAGAAGATT-3′<br>R: 5′-GGGCAGTAGCGAAGCAGAAT-3′
IFITM1	F: 5′-CCAAGGTCCACCGTGATTAAC-3′<br>R: 5′-ACCAGTTCAAGAAGAGGGTGTT-3′
BGN	F: 5′-GGTGGTCTATCTGCACTCCAA-3′<br>R: 5′-GGCTGATGCCGTTGTAGTAGG-3′
EGFR	F: 5′-AGGCACGAGTAACAAGCTCAC-3′<br>R: 5′-ATGAGGACATAACCAGCCACC-3′
36B4	F: 5′-ATCCCTGACGCACCGCCGTGA-3′<br>R: 5′-TGCATCTGCTTGGAGCCCACGTT-3′
